# Time for Care: Male and Female Voices Based on Their Caregiving Experiences

**DOI:** 10.3390/healthcare12222245

**Published:** 2024-11-11

**Authors:** Claudia Andrea Ramírez-Perdomo, Claudia Patricia Cantillo-Medina, Alix Yaneth Perdomo-Romero

**Affiliations:** Faculty of Health Sciences, Nursing Program, Surcolombiana University, Neiva-Huila 410001, Colombia; claudia.cantillo@usco.edu.co (C.P.C.-M.); alixyaneth.perdomo@usco.edu.co (A.Y.P.-R.)

**Keywords:** caregivers, social construction of gender, gender role, chronic disease, qualitative research

## Abstract

**Background/Objectives**: To understand the lived experiences of the informal caregivers of people with chronic noncommunicable diseases and their interpretations of the significance of that care. The perspectives of men and women interacting with people in a situation of chronicity are examined. **Methods**: This qualitative, phenomenological, hermeneutic study was based on Van Manen’s comprehensive and interpretative vision. Criterion-oriented non-probability random sampling was used. In-depth interviews were used to collect the information. Twenty informal caregivers participated in the study. **Results**: The following five themes emerged: transformation of the caregiver’s world; uncertainty of care; tireless guardianship and unwavering devotion; isolation and emotional exhaustion; and imbalance between caring for others and the self. **Conclusions**: The experience of informal caregivers reveals that both men and women face significant challenges, albeit from gender-differentiated perspectives and experiences. Men and women elaborate different meanings of caregiving, reflecting their gender roles and expectations. Therefore, an inclusive and equitable approach that recognizes and addresses these gender differences is essential in order to improve the support and quality of life for all informal caregivers, respecting and valuing their unique voices and experiences. An inclusive and equitable approach that recognizes gender intersections is required to improve support and the quality of life for informal caregivers.

## 1. Introduction

By 2050, more than two million people worldwide will be over 60 years of age [1]. These demographic changes will cause an increase in chronic noncommunicable diseases (CNCDs) such as cardiovascular and respiratory diseases, diabetes, cancer, and mental disorders, which are already major causes of illness, death, and disability in the Americas [1,2]. Moreover, mental disorders, substance use, injuries caused by traffic accidents, and interpersonal violence can lead to disabilities [2].

Disability limits the equal participation of people in society [3] and increases the demand and need for health services and long-term care [4], owing to the dependence that disabilities generate. In most cases, this care is provided by a family member who does not receive remuneration [5,6]. Chronicity, which is characterized by the progression of disease over time and its inherent complications, gives rise to a situation of dependence, which in turn necessitates the presence of a caregiver.

The care provided by informal caregivers involves helping with transportation, preparing food, cleaning the house, washing and mending clothes, providing personal care, administering treatment, performing medical procedures, scheduling, managing care actions, and taking care of financial activities [6]. The care of people in a situation of dependency is mostly related to poverty and is considered a private problem that must be solved by the family; it is a task that is mainly delegated to the women of the family [7,8].

Historically, women have been considered responsible for caregiving tasks because of their nature and gender-cultural influences [8,9]. These influences guide women to take care of their children or parents out of moral responsibility or debt; in addition, they face strong social pressure to take on the role of caregiver [1]. Thus, the feminization of care persists. The care of sick people is delegated to women because of their status as mothers, wives, or daughters, which makes them responsible for these tasks and their costs. This responsibility limits their social, economic, and affective development in the public sphere, which then perpetuates the gender inequalities that endure today [10].

Changes are occurring in the caregiver landscape for older adults; the number of men taking on the role of caregiver has increased in recent years [11,12,13]. Despite this change, a greater proportion of women continue to exercise the role of caregiver, compared to men [11,13]. These differences in representation between men and women in caregiving are due to gender stereotypes that consider caregiving as a ‘feminine issue’ and a ‘feminine-type’ activity [14]. In the context of Latin America, caring is considered an innate quality of women, deeply bound up with the traditional cultural phenomenon of Marianismo [15].

However, male informal caregivers also face significant pressures. For example, male informal caregivers experience greater social distancing than women [16], which negatively impacts their emotional and psychological well-being. In addition, men who take on the role of informal caregivers often face pressure to challenge gender stereotypes with the need to find a balance between caregiving and maintaining their masculine identity [17,18], which can lead to stress and feelings of isolation.

The understanding of how men approach caregiving, their coping mechanisms, and how this influences their identity and self-concept is still not as well understood as for women informal caregivers. The entry of men into what has traditionally been considered a predominantly female field should guide the understanding of how masculinities are constructed and carried out by men in caregiving tasks [13]. Although men represent a minority among informal caregivers [19], promoting greater male participation in informal care is necessary.

Breaking the roles traditionally assigned to women in care may be the change necessary for eliminating gender inequalities, which could in turn improve the quality of life of dependents, informal caregivers, and their families [20]. Male informal caregivers are increasingly recognizing the importance of their roles in providing emotional and practical support to their loved ones, which is challenging the entrenched gender norms in society [16].

Caring for people with chronic diseases negatively affects women’s health and well-being, as women represent the majority of informal caregivers. They experience greater stress and face worsening economic situations, resulting in poorer physical and psychological health compared to male informal caregivers [8]. However, male informal caregivers also experience particular challenges related to the increased effort to maintain their identity by adhering to masculine norms with a protective approach to caregiving, and a reluctance to seek help or talk about their emotions, leading to social distancing which negatively affects their lives [17]. It is essential to consider gender differences in caregiving experiences to promote inclusion and equity of informal caregivers. The knowledge gap stems from the lack of peer-reviewed studies that address gender differences in informal caregivers of people with CNCDs, taking into account that the number of men who assume the role of informal caregiver has increased in the population.

This study collates and evaluates the voices of men and women who have taken on the responsibility of caregiving. We examine the tapestry of experiences that combine love and commitment. We explore the experiences of mothers, sons, daughters, wives, and husbands who have transformed their lives to care for loved ones who are in a situation of chronicity and exhibit a high degree of dependence; these are people who have taken on the caregiver role with strength and determination. Therefore, we propose a phenomenological, hermeneutic, qualitative study aiming to understand the lived experiences of informal caregivers of people with CNCDs, and the meanings of care developed from the perspective of men and women, during their interactions with people afflicted by chronicity.

## 2. Materials and Methods

### 2.1. Design

Following van Manen’s methodology, this qualitative study adopted a phenomenological hermeneutic design aiming to describe, understand, and interpret the participants’ experiences of caregiving [21,22]. Phenomenology allows us to describe lived experience; hermeneutics makes it possible to understand how the individual interprets the texts of life; and, finally, semiotics allows us to develop a practical and written linguistic approach to phenomenology and hermeneutics.

### 2.2. Study Setting and Recruitment

The study population consisted of informal caregivers of people with CNCDs, as registered in the Care and Caregiver Support Project database, which is maintained by the nursing program of Surcolombiana University, Colombia.

Criterion-oriented, non-probability random sampling was used to review and study cases that met the predetermined criteria; this strategy seeks to ensure high data quality [23]. People who provided clear information related to their experiences were selected, which allowed for the potential manifestation of the experiences and increased the quality and quantity of information they provided for interpretation.

In phenomenological hermeneutic research, the number of informants is not considered significant. Instead of talking about a sample, reference is made to obtaining ‘examples’ of enriching descriptions of the experience [24]. The sample size is not predetermined; what is considered important is the ability to represent the lived experiences of informal caregivers.

### 2.3. Inclusion and Exclusion Criteria

The sample was selected homogeneously [25] based on the following inclusion criteria: being over 18 years of age and being a caregiver for a period longer than three months, and for longer than three hours a day. Informal caregivers of people experiencing acute disease crises were excluded.

### 2.4. Data Collection

The data were collected between March 2022 and June 2023. Participants who met the inclusion criteria were selected from the program’s database. They were contacted by telephone and informed about the study’s objectives. If they agreed, a face-to-face interview was scheduled at a location chosen by the participants.

Before the interviews, the participants signed an informed consent form. The chosen data collection technique was in-depth interviews. According to Van Manen, this type of interview mainly seeks to gather material on the lived experience through which the interviewee reflects on the phenomenon under study; this allows for the exploration and collection of narrative experiential material, stories or anecdotes that serve as a resource for phenomenological reflection, thus developing a rich and deep understanding of a human phenomenon [21].

The interviews were conducted by the main researcher at the participants’ homes. The opening question was: “Tell me what has the experience of being an informal caregiver meant to you, as a man or woman?” This question made it possible to reflect in the conversational relationship on the meaning of the lived experience of all participating caregivers. The interviews were recorded, listened to, and then transcribed within 24 h. The transcriptions were conducted at the semantic level by research assistants, and the recordings were stored and safeguarded by the researchers. Anonymity was maintained by assigning random names chosen by the researchers (Beatrice, José, Olga, etc.).

The collection of information ended when it was considered that enough information had been obtained to allow the construction of enriched phenomenological texts. This consideration is made under subjective reasoning of completeness, since in phenomenology the existence of any algorithm, statistical criterion, or formula for determining data saturation [22] is unknown. Therefore, it makes no sense to talk about saturation in this approach [21,22].

### 2.5. Data Analysis

We used van Manen’s [20] data analysis proposal, which consisted of six methodological steps: collecting data on lived experiences; transcribing the interviews into text; reflecting on the experiences by reading and rereading the interviews and getting acquainted with the text; recovering the semantic language and paying attention to the language used by the participants; searching for common patterns; identifying emerging issues and the interconnections between them; and finally, writing about the lived experiences by highlighting the presence of the studied phenomenon. Three researchers independently analyzed the interviews as a triangulation strategy [26], then shared the results in focus groups, which allowed for the definition of the themes.

### 2.6. Ethical Considerations

This study was approved by the Ethics and Bioethics Committee of Surcolombiana University (Act No. 005) on 8 October 2021. The ethical principles of beneficence, non-maleficence, autonomy, and justice were also considered. The participants’ data were kept anonymous by assigning them fictitious names.

### 2.7. Rigor and Reflexivity

We followed the rigor criteria proposed by Lincoln and Guba [27]. Credibility and confirmability were achieved by returning the interviews to the participants and determining whether they felt they were represented accurately and fairly in the text. Transferability was achieved by creating conditions in which the results would be useful in other contexts too. Dependability was assured through the clarity of methodology, type of sampling, data collection, data analysis, and the construction of information-rich texts validated by the participants and external researchers. Reflexivity is a qualitative research strategy through which researchers construct, collect, select, and interpret data; the researchers’ presence is fundamental to this process [28].

## 3. Results

Twenty informal caregivers—twelve women and eight men—were interviewed. Men were informal caregivers of adults only (Table 1).

**Figure 1:**
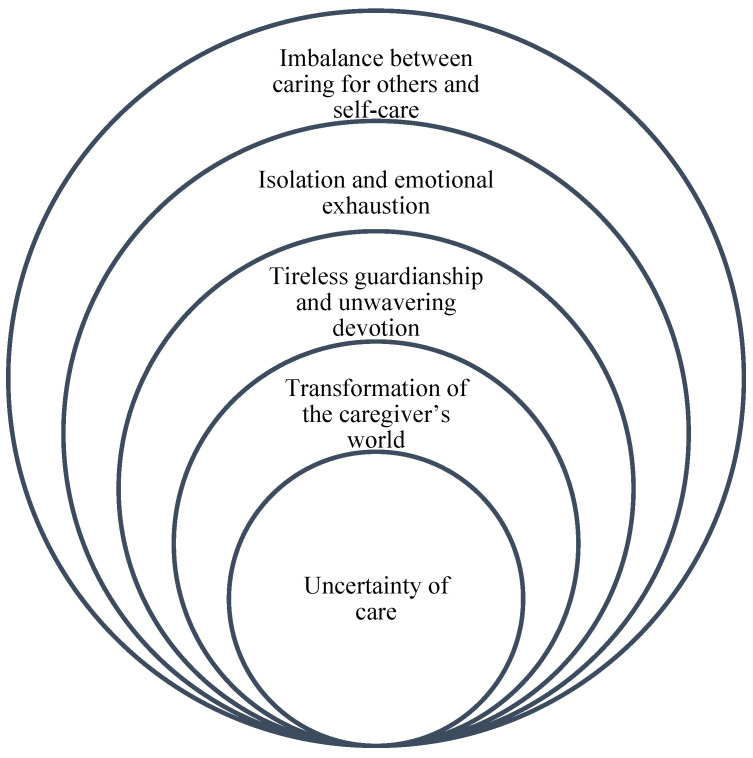
Experience of male and female informal caregivers (n = 20). Source: Created by the authors.

Analysis of the data yielded the following themes: uncertainty of care; transformation of the caregiver’s world; tireless guardianship and unwavering devotion; isolation and emotional exhaustion; and imbalance between caring for others and self-care (Figure 1).

Figure 1 symbolizes how each of the themes that emerged is interconnected and how one inevitably leads to the other. The uncertainty of caregiving is the basis that triggers a series of transformations and commitments that culminate in burnout and imbalance between caring for others and caring for oneself. This structure reflects the complex and multifaceted nature of informal caregivers’ experience, where each issue is not only a phenomenon in itself, but also part of a process that defines the totality of their experience.

### 3.1. Uncertainty of Care

Informal caregivers face harsh scenarios in light of their loved ones’ situations. It is not easy to assimilate reality; coping with the illness of loved ones without knowing how to take care of them is a complex and difficult challenge. The caregivers expressed that it was initially difficult to accept and understand, and that over time, they learned to better understand the patient and to communicate more assertively. They also expressed constant concern and a sense of insecurity about their loved one’s futures. The constant challenges and difficulties produced anxiety, fear of change, and feelings of emotional burden owing to the uncertainty associated with caregiving.

Women experienced a sense of uncertainty and constant anxiety. They used terms such as ‘anxiety’, ‘loneliness’, and ‘emotional exhaustion’ to describe their experiences. They highlighted uncertainty about the future, the feeling of being in limbo, and not knowing what would happen next.

“The hardest thing about taking care of a person is living with the anxiety that one day they may leave… We may go to bed one night and find they do not wake up the next day.”

(Olga)

“Ever since the beginning, I have felt uncertainty, loneliness, and permanent emotional exhaustion.”

(Camila)

“For me, at the beginning, I felt fear, restlessness and anxiety, not knowing what was going to happen.”

(Rocío)

“I was not used to dealing with a sick person like that… I cry; I sometimes sit down to cry about what has happened to me, that is, to have been left alone like this, to be with him like this.”

(Melba)

In contrast, the men adopted a more pragmatic and process-oriented stance, focusing on the need to gradually adapt and acquire knowledge about the disease as the caregiving process progressed. This attitude may reflect an objective problem-solving approach adopted by the men.

“At first you are trying to adjust to the situation, it is like a process. As you are not fully prepared to understand it, the adjustment is always a difficult step.”

(José)

“They (healthcare staff) only say ‘Give the drug to your mom like this or that’… and that’s it. No one explains anything further to us.”

(Pablo)

“Hemodialysis is too hard, sometimes you see difficulties in some things and if you go back… you don’t know what to do.”

(Raúl)

“I am worried about what will happen to my wife when I am no longer with her.”

(Jairo)

### 3.2. Transformation of the Caregiver’s World

The participants’ new roles imply adaptation and profound transformations of their lives, among which the necessity to develop emotional skills stand out—such as patience—to meet the needs of their loved ones, which often go beyond the provision of just physical assistance. Some informal caregivers described the internal conflict they often faced between the desire to give up owing to emotional exhaustion, and the responsibility and commitment required to take care of their loved ones. The latter is what drove them to keep going.

Women highlighted the emotional and physical impacts of caregiving on their lives. They expressed that they felt the constant burden of responsibility, the internal struggle between tiredness and dedication, and reorientation of priorities toward the well-being of the person being cared for. Their approach was emotional and subjective. They highlighted their personal challenges and the transformation of their worlds, based on the demands of caregiving.

“I do not rest from taking care of her, I have no rest… I have to keep an eye on her, do my job and keep an eye on the child, one forgets to take care of oneself.”

(Olga)

“One has to do more work; more dedication and effort is required on an emotional, economic, physical level, everything, everything… Sometimes you get up, and you do not want to start, you do not want to go on, but just seeing him… realizing how much he needs our care, that he needs us one hundred percent, you go back and start again.”

(Rocío)

“Everything has been a process that has been carried out gradually. You must have a lot of patience; if you do not have patience, it is difficult, and sometimes there are inconveniences.”

(Beatrice)

“Well, it changes everything because you do not think about anything else, you do not focus… you only think about the well-being of that person.”

(Mery)

Men adapted to the role of caregivers by developing patience and managing their emotional burdens. Although they experienced stress, they showed the willingness to learn and adjust to the demands of caregiving. They highlighted the need to remain calm and attentive to the needs of their loved ones.

“I think the most important thing is patience. That is what I was telling them: I had to change to become patient. I know that the patients have an attitude, you have to get used to it and adapt to that change, right? You must be patient to understand their situation.”

(Joseph)

“Well, it was difficult for me, but then you need to stand up and take on the burden.”

(Jaime)

“Patient with a sick person, that is what you should be, I think. Give them their medicines on time and pay attention to everything that happens.”

(Mario)

“For example, when I need to leave, the nurse stays. However, I cannot walk calmly, I get stressed. I am always stressed; sometimes I forget… but no, soon I stop doing other jobs… I have to go back to the stress of the illness.”

(Pedro)

“Well, I would go and pick her up, bring her back and she would go to bed, because she didn’t come in the mood for anything… to handle the stress that they handle, plus the bad temper that was added… I would make lunch, help to pick up the mess or something.”

(Raúl)

“I have to take care of everything she needs because I am responsible for her and that’s the way it should be, sometimes I get stressed because there are many things, and all of them are important, so I tell myself I need to be patient.”

(Jairo)

### 3.3. Tireless Guardianship and Unwavering Devotion

The informal caregivers expressed the need to be constantly attentive to their loved ones. This meant being available 24 h a day and adapting their routines to meet patients’ needs. The care involved vigilance and attention to the changes in the patient’s condition. They had to be alert even at night, which prevented them from getting a good night’s rest and led to stress, anxiety, and emotional exhaustion.

For women, care focused on the fragility and specific needs of the person in their charge. Caring was a responsibility that they took on with devotion; however, they did not necessarily see it as a moral obligation derived from the past, but as an expression of love and care toward a loved one who needed constant attention.

“I take constant care of her… I do not allow a third party to come and take care of my daughter, because she is fragile… first my daughter, then the rest, and even I can wait.”

(Olga)

“I get up, give him breakfast, his medications, I have to be very attentive; I have to give my son food. So now I am living in terrible chaos, I have no one to help me. I have dedicated myself only to taking care of J.”

(Ana)

Men perceived caregiving as a moral obligation. They felt that they could not abandon their parent/patient because of their gratitude for the love they received during their upbringing. Their commitment to caregiving was a way to give back what they received and to express their gratitude toward the person they cared for.

“She is my mom, and I have always been looking out for her, and at this moment, she needs me the most; how am I going to abandon her? Especially as she never abandoned us.”

(Pablo)

“Perhaps when I was little, when I was born, she went through similar situations with me… at the time, she did everything, now it is my turn to do it for her.”

(José)

“I became aware that I have to stay with him until God gives me life and health, and to have the hope that he will be transplanted.”

(Nicolás)

### 3.4. Isolation and Emotional Exhaustion

Caregivers expressed feelings of emotional and physical exhaustion, social isolation, giving up other activities, and a constant psychological burden in their day-to-day lives, which negatively affected their quality of life and triggered depression and anxiety. Their social lives were affected by the demands of caregiving. They could not participate in social or leisure activities, and often sacrificed their social lives to ensure proper care.

The women highlighted the emotional exhaustion and sense of isolation they experienced as caregivers. They expressed an intense emotional burden and a sense of loneliness in their caregiving experience, as well as a lack of external support to cope with it.

“She is the priority, well, the truth is that I do not have a lot of time for myself; my priority is my daughter, as long as she is well, I am well…”

(Berta)

“The only thing I go out for is medical appointments. I cannot say, ‘Oh, I am going to a shopping mall this afternoon, to hang out, to window shop’… I cannot… you cannot go out.”

(Rocío)

“The caregiver works in solitude, a solitude that we are dragged into by the journey that people with Alzheimer’s make; we carry anguish, sadness, depression, a heavy suitcase that no one helps us carry.”

(Beatrice)

Men mentioned the stress and physical fatigue associated with caregiving, and their concerns about leaving the person in their care alone. Their lives were centered around the practical responsibility of caregiving, which limited their ability to enjoy activities outside the home.

“You focus on the sick person… stress, and that makes you tired even if you are sitting there looking at him lying there.”

(Mario)

“If I go out, she stays alone; because at these moments, I am thinking about it; because I leave her in a chair and she stays there, and because she does not sit up, you cannot go out.”

(Felipe)

“I am tired and sick but I have to keep an eye on her, she was an excellent wife, now it is up to me to take care of her.”

(Jairo)

### 3.5. Imbalance Between Caring for Others and Self-Care

These excerpts show how informal caregivers face an imbalance between dedicating themselves to the care of their loved ones and attending to their own needs and desires. Responsibility toward others often takes precedence over self-care.

Women neglected their needs and desires, prioritizing the needs of their loved ones. Their identities and independence were often subordinated to their roles as caregivers, which led to an imbalance between caring for others and themselves.

“First comes my daughter. Sometimes, I ask myself the same thing: one forgets about being a woman and a person, when one is mother to a child with a disability or a person with a disability; sometimes I need to go out but do not, because she comes first.”

(Olga)

“I take a back seat, my tiredness and pain are not important, it is not that it does not hurt. However, I care more about taking care of my mother; she always needs me, and I can wait, I can handle this.”

(Luisa)

Men mentioned the challenge of balancing their own needs and desires with the demands of caregiving. Although they expressed the importance of caring for the person in their charge, they also mentioned the necessity of meeting their own needs for rest and well-being.

“You have to stop doing personal things for the good of the person, because what matters is the person. However, the situation in which we both live is complicated.”

(Philip)

“I come home tired from work, wanting to sleep, but then I find her unwell; I have to… try to distract her, but I also want to go to sleep; so it’s these things that make me feel guilty. I see it, but I can’t do anything about this situation.”

(Jaime)

## 4. Discussion

The literature shows that the burden of care falls predominantly on women [1,7,20,29,30], resulting in gender inequalities in healthcare that continue to grow [31]. These inequalities occur in addition to other societal gender disparities. It is crucial to recognize and address these gender inequalities in the context of informal care, which is becoming increasingly necessary in the present day [7], and to promote equity in health and society in general [20]. The social constructs of masculinity influence men’s caregiving roles in society. Male identities are shaped by the social and cultural expectations of caregiving and parenting. These constructions impact gender equality and highlight the importance of challenging entrenched gender stereotypes to promote meaningful change toward a more equitable society [32].

In the present study, men and women experienced uncertainty differently. Women experienced anguish, suffering, and uncertainty about the future, whereas men acquired knowledge to manage the disease and adapted gradually. Rabiei et al. [33] established that informal caregivers experienced frustration and felt like they were being held captive by disease-related complications. They faced difficulties in coping with them. Tolerance of the permanent suffering of the patient increased the caregivers’ difficulties and frustration. In addition to feelings of inferiority, worry and uncertainty about the future are common to caregivers from all cultures, especially in the context of medical care, which makes relatives and caregivers feel captive to the disease; many surrender to it.

Informal caregivers perceived that their worlds had been transformed: women because of the burden of care, fatigue, and emotional and physical impacts, and men because of stress. However, men learned to manage their stresses and respond to care needs. In chronicity, the lives of caregivers and sick people change because of the pragmatic adjustments they must make in their lives; they implement strategies that allow them to face the disease and manage the burden of caregiving [34].

Women feel physically and emotionally exhausted by their caregiving roles, which highlights and reinforces traditional female stereotypes about caregivers and limits the possibilities of pursuing their interests, personal needs, professional ambitions, sexual identity, and social status. In turn, male caregivers may block some emotional aspects of caregiving, suppress emotions, and restructure their caregiving roles. When this is achieved, they experience a sense of honor in that success and a sense of self-management [35].

Women provide care with unwavering will, focusing on the patient’s condition of fragility with devotion, and as an expression of love. Men take care of their loved ones as a moral obligation and out of feelings of gratitude. Caregiving roles make women feel morally obligated to ‘do their duty’, but they can also provoke feelings of guilt when those expectations are not met [1]. This may be related to subordination to others, since, according to the Marianism theory, Latin women must show obedience and respect for traditional hierarchical gender structures and follow imposed behavioral norms [15].

For men, caregiving entails practical and emotional support; this is often manifested as ‘caring for’, which relates to caregiving through the performance of tasks, and ‘being concerned about’, which refers to the emotional aspects of caregiving. These may include the generalized relational and affective elements of ‘being supportive’ [13]. Men’s power and control are normalized [15], and they retain their role as providers and power in the area of care.

Caregivers’ isolation and exhaustion are related to their 24 h dedication to care. Women feel that their lives are complicated in such a way that they cannot perform other activities of daily living or leisure [7,36] because they must always dedicate themselves to their loved ones. They recognize that playing the role of caregiver causes a greater emotional and physical burden [7] because they sacrifice their time and their own needs for the well-being and joy of the person they care for; their dedication is unconditional. Women in the Latin context tend to remain silent in order to maintain harmony within the family, this leads them to repress their thoughts, beliefs, and personal needs in order to maintain the family unit at the expense of their own well-being [15].

Men experience stress and fatigue, their lives are centered around care, and their outings are restricted. The role of caregivers makes them relinquish their old jobs and being providers of their homes, having conversations with friends, and solving problems; they simultaneously suffer economic losses and experience a lack of future projects to look forward to [37].

For both men and women, the transition to the role of a caregiver causes anguish and fear. Without knowledge of how to exercise their new roles, they fear not being able to meet the demands of care. They learn to provide care over time [36], and their levels of stress and anxiety about their experiences increase [38]. This fear of making mistakes affects the caregivers’ mental and emotional health.

Caregivers also experience intense distress when forced to leave those they care for alone [38]. The fear of mishaps or emergencies occurring during their absence creates constant concern for the well-being of loved ones. Sometimes, they feel guilty for being absent, which can generate internal conflicts between care responsibilities and personal needs.

The literature points to caregivers’ difficult task of balancing their responsibilities while providing care. Female caregivers are unaware of their own needs and desires, and they often sacrifice their time, energy, and personal aspirations to ensure the well-being of the person they care for. In contrast, men try to maintain a balance between their needs and the demands of care. Both men and women who are informal caregivers experience a loss of direction in their lives, leisure time, and life projects [37]. Being a caregiver produces negative effects that are aggravated by the differences between the two sexes. In general, women are more bound to their homes, have less leisure time, and sleep less than men [19].

## 5. Conclusions

As informal care continues to be the backbone of care for people with dependence, it is important to explore the experiences and meaning of caregiving from a gender perspective and recognize the intersections of gender and care. This will allow us to move toward a future in which care is inclusive, equitable, and empowered. The results of this research allow us to identify the essential differences in care, such as the way in which each care provider faces uncertainty; the substantial differences between men and women when facing life changes; the perspective assumed by each gender in the tireless and devoted roles of one and the other, with a marked influence of the Latin patriarchal system; the experience of burnout; and, finally, giving up on self-care in order to concentrate on caring for others.

### 5.1. Strengths and Limitations

The strength of this research lies in exploring the aspects that differentiate male and female informal caregivers when taking on the challenge of caring for a person with a chronic condition. The limitations pertain to the type of sampling employed and the inability to generalize the results.

### 5.2. Recommendations for Further Research

We suggest that primary care, especially nursing, can promote a change in the traditional model of care, from its privileged position of proximity to people and the community. To achieve this, it is crucial to integrate the gender perspective in nursing practice and in the research and interventions aimed at caregiver care. It is also important to introduce collaborations between the state, society, and families. It is important to develop a gender-focused nursing theory to address informal care in such a way that it responds to the demands specific to this area.

### 5.3. Implications for Policy and Practice

The provision of appropriate care for families with a dependent member requires an understanding of the complexity and uniqueness of each family system. It is essential for professionals to have a thorough understanding of this dynamic to offer the most effective support. This involves working closely with the family to identify and prioritize their needs and ensuring that interventions are tailored to their specific requirements, which could then lead to an improvement in the quality of family life. Similarly, public health policies must be guided by a process of understanding informal care from a gender perspective, to address the needs and differences between men and women who assume that role, aimed at improving protection actions for this population that cares and learns to care in solitude.

## Figures and Tables

**Table 1:** Sociodemographic characteristics (n = 20).

Caregiver	Sex	Age (Years)	Relationship	Diagnosis of the Person Cared for	Age of the Person Being Cared for (Years)
José	Male	53	Mother	Alzheimer’s disease	70
Mario	Male	78	Wife	Hypertension and diabetes	72
Berta	Female	45	Daughter	Psychomotor retardation	19
Felipe	Male	55	Wife	Paraplegia and muscular dystrophy	47
Olga	Female	32	Daughter	Ataxia	10
Rocío	Female	49	Husband	Stroke COVID-19 sequelae	60
Pedro	Male	47	Mother	Chronic renal failure	72
Jaime	Male	34	Wife	Bipolar disorder	44
Luisa	Female	56	Mother	Alzheimer’s disease	80
Camilla	Female	56	Father Mother	COPD Alzheimer’s disease	87 82
Lupe	Female	60	Daughter	Cognitive disability	22
Mery	Female	25	Mother	Bipolar disorder	50
Ana	Female	50	Son	Schizophrenia	29
Melba	Female	58	Husband	Head trauma, multiple neurological injuries	62
Nicolás	Male	50	Father	Chronic renal failure	18
Gladys	Female	24	Son	Hydrocephalus, musculoskeletal malformations	8
Beatrice	Female	38	Son	Duchenne muscular dystrophy	15
María	Female	61	Son	Cerebral palsy, spastic quadriplegia	31
Raul	Male	50	Wife	Chronic renal failure	32
Jairo	Male	86	Wife	Alzheimer’s disease	83

Legend: COVID-19, coronavirus disease; COPD, chronic obstructive pulmonary disease. Source: Created by the authors.

## Data Availability

The data supporting this study’s findings are available on request from the corresponding author. However, due to privacy or ethical restrictions, the data are not publicly available.
